# Feasibility of dose accumulation for assessing treatment-related side effects in MR-guided abdominal SABR: A single-institution study

**DOI:** 10.1016/j.ctro.2026.101277

**Published:** 2026-09-02

**Authors:** Rekaya Shabbir, Agnieszka Zakacz, Ruksana Sivakaran, Eliana Vasquez Osorio, Marianna Theodoulou, Zainul Kapacee, Ananya Choudhury, Laura J. Forker, Ganesh Radhakrishna, Cynthia L. Eccles, Mairead Daly

**Affiliations:** a Division of Cancer Sciences, Faculty of Biology, Medicine and Health, University of Manchester, Oxford Rd, Manchester, M13 9PL, UK; b The Christie NHS Foundation Trust, Wilmslow Road, Manchester, M20 4BX, UK

**Keywords:** MR-guided radiotherapy, Adaptive Radiotherapy, Side effects, deformable image registration, Abdominal radiotherapy, hepatocellular carcinoma, pancreatic cancer, SABR

## Abstract

**Background:**

Daily adaptive magnetic resonance (MR)-guided treatment changes delivered dose distributions, making cumulative dose uncertain. This work assessed the feasibility of exploring potential relationships between side effects and accumulated dose (AD) in MR-guided stereotactic ablative radiotherapy (SABR) for liver and pancreatic cancers. Using deformable image registration (DIR), AD to abdominal organs at risk (OARs) were estimated and potential relationships with observed side effects explored.

**Methods:**

Forty-one patients treated with 5-fraction MR-guided SABR to primary or metastatic pancreatic and liver tumours were retrospectively included. Daily adapted plans were deformably mapped and summed to estimate AD. Differences between accumulated and planned dose were assessed. Changes in clinician-reported symptoms collected at baseline, 3, and 6 months were analysed. Associations between AD and symptoms were assessed using Spearman's rank test.

**Results:**

Most AD metrics were lower than planned for gastrointestinal OARs, reflecting potential differences from delivered ATS plans, with significant reductions in duodenum and large bowel D0.5cc and D10cc (*p* < 0.001). Weak associations between small bowel dose metrics and acute and subacute diarrhoea were found (r_s_ 0.4–0.5, *p* < 0.040). All side effects were ≤ grade (G) 2. Median overall survival for locally advanced pancreatic cancer (LAPC) patients was 16.0 months post-SABR and was not reached for other subgroups.

**Conclusion:**

Exploring potential relationships between DIR-based accumulated OAR dose and side effects was feasible. Preliminary relationships between accumulated OAR dose and side effects were observed; however, further work in larger studies is warranted before these findings can be used to personalise treatment in the future.

## Highlights

• DIR-based dose accumulation was feasible in MR-guided abdominal SABR.
• Accumulated OAR doses were typically lower than planned.
• Small-bowel dose correlated with acute diarrhoea after SABR.
• Large-bowel dose correlated with subacute abdominal pain.
• Demonstrates feasibility of exploratory dose–toxicity analysis in MR-Linac.

## 1 Introduction

Outcomes for patients with hepatobiliary-pancreatic (HPB) cancers remain poor. In England, one-year survival is 7% for pancreatic and 34% for primary liver cancer [1], [2], while global incidence is projected to double over the next 40 years [3]. The pancreas and liver are also common metastatic sites, including from renal cell carcinoma (mRCC) [4], and colorectal cancer (mCRC) [5]. Although surgery is standard for limited-stage disease, 90% of pancreatic cancers are unresectable at diagnosis [6]. Radiotherapy has an expanding role in HPB cancers, but delivered dose remains poorly characterised and its relationship to outcomes and side effects is unclear.

Stereotactic ablative radiotherapy (SABR) is a promising option for patients with hepatocellular carcinoma (HCC) awaiting liver transplant, or for inoperable locally advanced pancreatic cancer (LAPC), delivering high doses in a few fractions while minimising dose to surrounding healthy tissue [7]. However, abdominal SABR is challenging because radiosensitive organs at risk (OARs), including stomach and small bowel, often lie close to the target [8]. Excess dose to gastrointestinal (GI) OARs can cause nausea, diarrhoea, ulceration and perforation [7].

The magnetic resonance linear accelerator (MR Linac) combines a linear accelerator with diagnostic magnetic resonance imaging (MRI), improving soft-tissue visualization compared with x-ray imaging and enabling daily plan adaptation to daily anatomical variation [9]. Consequently, accumulated dose (AD) over treatment may differ from the planned dose [10]. Dose accumulation enables assessment of these differences and their potential impact on outcomes [10]. Deformable image registration (DIR) facilitates non-rigid alignment between reference scan and daily scans, enabling dose warping and summation [11], [12].

This study aimed to quantify differences between planned dose and AD in patients undergoing SABR on the MR Linac, and to establish the feasibility of an exploratory methodological framework for evaluating relationships between AD and clinical outcomes.

## 2 Materials and methods

### 2.1 Patients, imaging and treatment

Forty-one consecutive patients treated with 5-fraction liver and pancreatic SABR between May 2022 and June 2025 on the Unity MR Linac (Elekta, Crawley, UK) were retrospectively included. Most patients (*N* = 30) had pancreatic targets. Patients provided informed consent through the ethics-approved, non-interventional MOMENTUM registry study (NCT0407530, [13]). Patients were fasted, positioned supine with arms raised, and immobilised with a full-body vacuum bag (Klarity Medical Products, Heath, Ohio, USA) and abdominal compression belt (Freedom X™, CDR Medical Systems, Calgary, Canada).

Four-dimensional computed tomography (4DCT) and contrast-enhanced three-dimensional (3D) computed tomography (CT) in exhale-breath hold (EBH) were acquired. Planning MRI (pMRI) was acquired on the MR Linac on the same day for reference plan (RefPlan) generation. The gross tumour volume (GTV) was delineated on exhale and inhale 4DCT phases by a clinical oncologist. For pancreatic cases, involved peritumoral lymph nodes were delineated separately where present, without elective nodal irradiation. For liver metastases, a 5 mm clinical target volume (CTV) margin was applied to the GTV to account for potential microscopic disease extension [14]. Structures were rigidly propagated to the pMRI. The internal target volume (ITV) was then generated to account for motion, expanded by 5 mm for the planning target volume (PTV), and then cropped 5 mm from PRVs. RefPlan was normalised to the centre of the cropped PTV, with 95% receiving 30–60 Gy in five alternate-day fractions, following national guidelines [14], [15]. A 5 mm isotropic planning organ-at-risk volume (PRV) margin was applied to GI OARs. Details are summarised in Supplementary Fig. 1.

Daily step-and-shoot intensity-modulated radiotherapy (IMRT) was delivered using the ‘adapt to shape’ (ATS) workflow. A fraction MRI (FxMRI) was acquired using identical parameters to pMRI. Manual rigid registration to pMRI was performed on the target using Monaco® (V5.51.11, Elekta AB, Stockholm, Sweden). OARs were deformably propagated, while the ITV was rigidly propagated because re-estimation of daily motion was unavailable; no new ITV was generated at each fraction. Initially, OARs within 2 cm of the PTV were manually edited by the oncologist or trained radiographers; this was later reduced to 1.5 cm. PRVs were re-applied and the PTV re-cropped away from PRVs. Daily adapted plans (ADT) were re-optimised with OAR constraints prioritised.

### 2.2 Dose accumulation

OARs were re-delineated throughout the treatment volume by a single trained observer (MD) for consistency. OARs varied by treatment site and patient anatomy; oesophagus, heart and chest wall were assessed for liver cases only. Doses were accumulated using the scripting interface in a research version of Raystation (V12, RaySearch Laboratories, Stockholm, Sweden). Briefly, hybrid intensity and contour-based DIR using the anatomically constrained deformation algorithm (ANACONDA) was performed following organ-wise rigid gray-level frame of reference (FOR) registration. Structure-specific deformation vector fields (DVFs), constrained by individual OAR and GTV contours, were generated, and ADT doses deformably mapped using the corresponding DVF for each structure prior to summation. Dose mapping and accumulation were performed on the reference dose grid (2 mm resolution), with DVFs calculated at 0.5 mm spatial resolution.

DIR quality assurance (QA) was assessed using mDTA and maximum Hausdorff Distance (HD) between original/deformably mapped contours across all patients and fractions, recognising that these metrics assess contour agreement rather than voxel-level deformation accuracy. Dice similarity coefficient (DSC) was also calculated for comparison with published literature, despite recognised limitations related to the structure volume dependence and limited spatial information [16]. Target registration error (TRE) was not assessed because reproducible anatomical landmarks were lacking within included OARs and imaging field of view (FOV) varied across fractions.

Differences between planned (RefPlan) dose and accumulated OAR doses were recorded. Near-maximum and volume-based metrics commonly reported in the literature were selected to enable comparison with previous studies [17], including mean dose (Dmean), dose to 0.5 cc (D0.5cc), 5 cc (D5cc) and 10 cc (D10cc) for GI OARs. GTV AD was evaluated using Dmean, minimum dose (Dmin), and dose to 95% (D95%) to contextualise OAR dose findings.

### 2.3 Symptom and outcomes assessment

A research nurse prospectively collected and graded acute and subacute side effects according to the Common Terminology Criteria for Adverse Events (CTCAE, V5.0) as part of the MOMENTUM study. Survival status was retrospectively extracted from the electronic patient record (EPR) up to the data cut-off of 5 February 2026, and was calculated from end of treatment until death or censored for patients alive at that date. Side effects and subsequent systemic or local treatments occurring within 6 months of SABR were extracted. Because this work aimed to assess the feasibility of identifying relationships between DIR-based AD and side effects, survival and subsequent treatments were summarised descriptively and were not included in the dose-symptom analysis. Hepatotoxicity was assessed using serial liver function tests, imaging, and clinical review.

### 2.4 Analysis

**Fig. 1:**
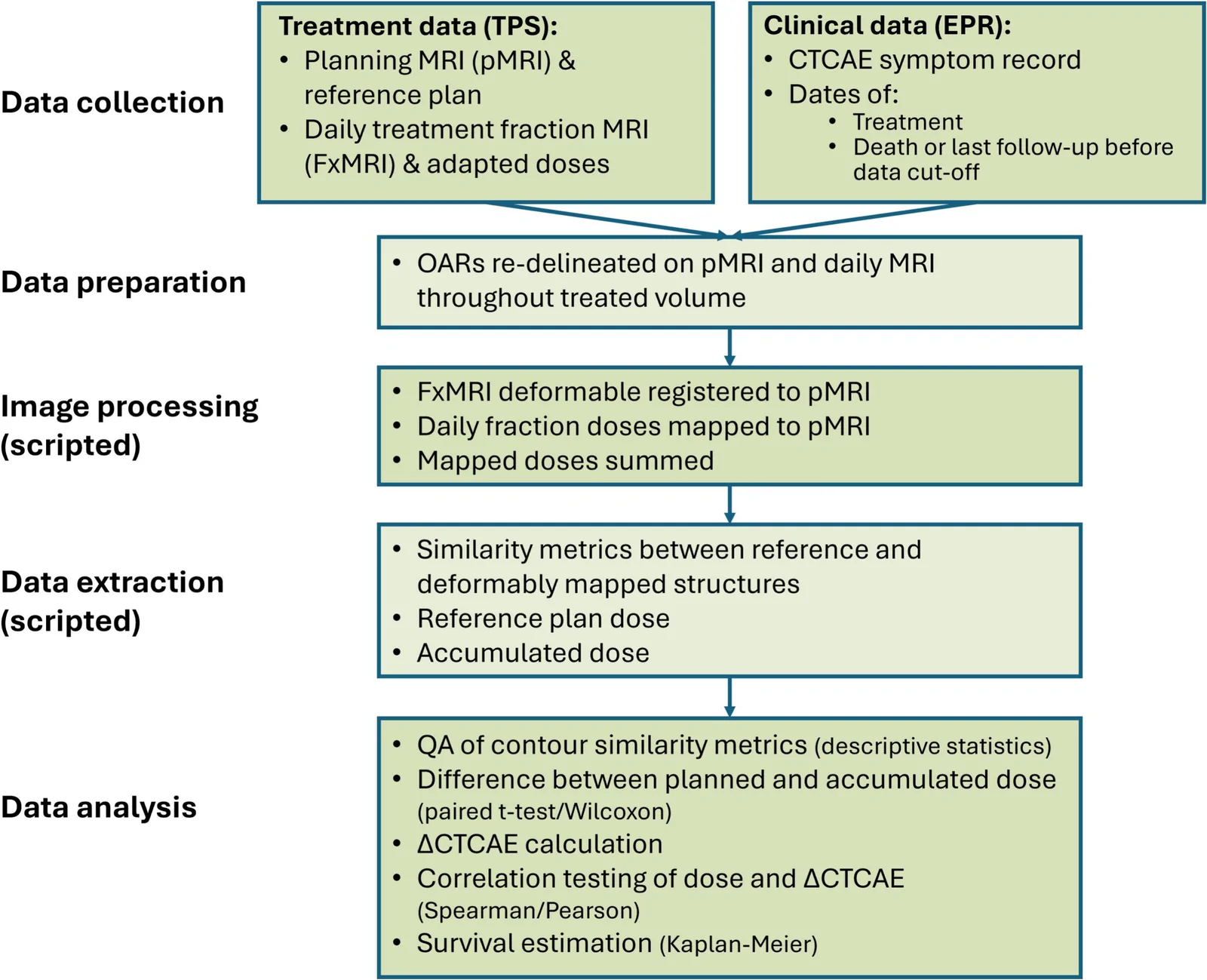
Summary of the workflow for deformable registration-based dose accumulation process, including data collection, re-delineation of organs at risk (OARs), deformable image registration between fraction (Fx) and planning (p) magnetic resonance imaging (MRI), including dose warping and summation, quantitative data extraction, and statistical analysis. EPR = electronic patient record. CTCAE = Common Terminology Criteria for Adverse Events. QA = quality assurance. ΔCTCAE = difference in CTCAE from baseline.

A structured pipeline was implemented comprising DIR with geometric QA, organ-specific dose mapping, and outcome association analysis (Fig. 1). Contour similarity metrics were summarised descriptively. Planned dose and AD were recorded, plotted in Excel (V2507, Microsoft Corporation, Redmond, USA), and summarised descriptively. Comparison with summed ATS plan doses was not performed. Normality of paired dose differences was assessed using the Shapiro-Wilk test in Prism (V10.2.2, GraphPad Software Inc., Boston, USA). Differences where *p* > 0.05 were analysed using paired *t*-tests, and those where *p* ≤ 0.05 with the Wilcoxon signed-rank test. A two-sided significance level of *p* < 0.05 was used.

Symptom changes from baseline were calculated per patient (ΔCTCAE) at 3 and 6 months. Univariable associations between AD metrics and ΔCTCAE were explored using Spearman's rank correlation in Prism. Multivariable analysis was not performed due to the limited number of symptom events. Median overall survival (OS) was estimated using the Kaplan-Meier method in Prism [18]. Survival was included descriptively for clinical context only, rather than as definitive disease-control or dose–outcome endpoints. Given the heterogeneity in primary histology and small subgroup sizes, associations between accumulated target dose and oncological outcomes were not analysed.

## 3 Results

### 3.1 Patients

Treated lesions included primary tumours and metastases within the pancreas or liver only. Neoadjuvant chemotherapy was administered in 23/25 patients with LAPC (Table 1). All 11 patients with liver targets were Child-Pugh score A, and three had received trans-arterial chemoembolization (TACE) ≥ 3 months prior to SABR. Involved peritumoral lymph nodes were included in the target volume for 2/30 pancreas patients (6.7%). One patient referred from an external centre was lost to follow-up and therefore censored from survival evaluation due to missing data.

**Table 1:** Characteristics of included patients. TACE = trans-arterial chemoembolization, Gem = gemcitabine. Gem-Cap = Gemcitabine-Capecitabine combination, FOLFIRINOX = folinic acid, 5-fluorouracil, irinotecan, oxaliplatin combination. mFOLFIRINOX = modified FOLFIRINOX regimen with dose reduction to improve tolerability. Gy = Gray. cc = cubic centimetres. Note: subsequent systemic treatment is within 6 months of SABR completion only.

		*n*	*%, SD, range*
**Tumour Location (n, %)**			
Pancreas		30	73.2
Liver		11	26.8
**Diagnosis (n, %)**			
Pancreatic adenocarcinoma		25	61.0
Metastatic renal cell carcinoma		5	12.2
Hepatocellular carcinoma		7	17.1
Intrahepatic cholangiocarcinoma		1	2.4
Metastatic colorectal cancer		2	4.9
Metastatic breast cancer		1	2.4
**Sex (n, %)**			
Male		23	56.1
Female		18	43.9
**Age (Mean, SD)**			
Years		66.2	8.2
**Prior local therapies (liver, n, %)**			
	TACE	3.0	7.3
**Neoadjuvant systemic treatment (pancreas, n, %)**		23	56.1
*Gem*		*4.0*	*17.4*
*Gem-Cap*		*2.0*	*8.7*
*FOLFIRINOX*		*10.0*	*43.5*
*mFOLFIRINOX*		*7.0*	*30.4*
**Prescribed SABR dose (Median, range)**			
Gy		40.0	30.0–60.0
**Subsequent systemic treatment post-SABR (n, %)**		4.0	9.8
*Gem*		*2.0*	*50.0*
*Gem-Cap*		*1.0*	*25.0*
Elacestrant/densumab		*1.0*	*25.0*

### 3.2 Planned versus accumulated dose

**Fig. 2:**
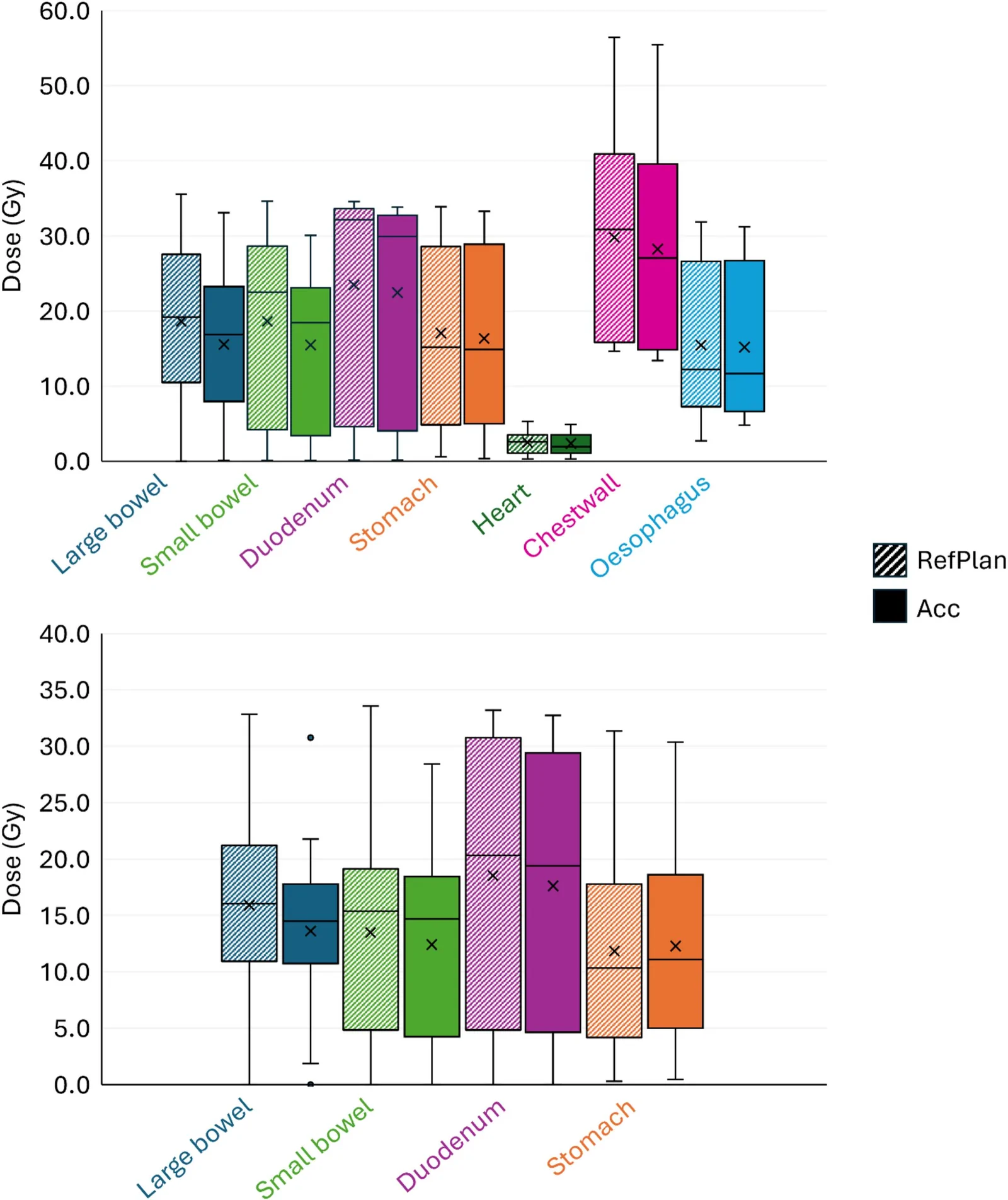
Box-and-whisker plot showing differences in planned (shaded) and accumulated (solid fill) doses to 0.5 cc (D0.5cc, top) and D10cc (bottom) for organs at risk (OARs).

Good geometric accuracy was found for all deformably-mapped OARs, with full results (including mDTA, HD, and DSC) reported in Supplementary Table 1. Accumulated OAR doses were typically lower than planned (Fig. 2), with statistically significant differences for several structures and parameters. Accumulated GTV Dmin was significantly higher than planned (median difference 0.6 Gy, *p* = 0.005, Table 2), while D95% was not (*p* = 0.471). Accumulated D0.5cc and D10cc for large and small bowel, and duodenum, were significantly lower than planned (*p* < 0.001). Other accumulated OAR doses were typically lower than planned, although not statistically significant.

**Table 2:** Comparison of dose metrics between the reference plan (RefPlan) and accumulated dose (Acc Dose) across structures (*N* = 41 patients). Data are presented as medians with interquartile ranges (IQR) in Gray (Gy). The median differences with corresponding IQR are calculated from paired per-patient differences, and *p*-values from statistical tests are also shown. Differences were calculated as Acc Dose – RefPlan, therefore negative values indicate higher AD than planned, and statistically significant differences (*p* ≤ 0.05) indicated with an asterisk (*). Note that patient numbers varied by structure availability, treatment site and patient anatomy; oesophagus, heart and chest wall were assessed for liver cases only. GTV dose metrics are reported for context only where a GTV structure was available.

(N)	Metric	Ref Plan (median/IQR)	Acc Dose (median/IQR)	Difference (median/IQR)	*p*
**GTV (*n*** **=** **35)**	Dmin	31.5 (30.2 to 36.0)	32.0 (30.7 to 33.8)	−0.6 (−1.1 to −0.3)	0.003*
	Dmean	41.9 (38.4 to 45.4)	41.0 (34.3 to 44.6)	0.3 (0.0 to 1.2)	0.002*
	D95%	35.2 (32.4 to 42.1)	33.9 (32.5 to 41.0)	0.1 (−0.6 to 0.7)	0.471
**Liver (*n*** **=** **41)**	Dmean	3.3 (1.6 to 7.8)	3.5 (1.7 to 7.4)	0.0 (−0.2 to 0.4)	0.522
**Large bowel**	D0.5cc	19.2 (10.8 to 27.6)	16.9 (8.1 to 23.1)	1.7 (0.5 to 4.5)	<0.001*
**(*n*** **=** **39)**	D10cc	16.0 (11.4 to 21.0)	14.5 (11.1 to 17.8)	1.6 (0.1 to 3.4)	<0.001*
**Small bowel**	D0.5cc	22.5 (5.4 to 28.6)	18.5 (4.9 to 22.9)	2.2 (0.1 to 5.9)	<0.001*
**(*n*** **=** **40)**	D5cc	17.0 (7.1 to 21.2)	15.8 (7.0 to 19.6)	1.1 (0.0 to 4.0)	<0.001*
	D10cc	15.4 (5.7 to 19.0)	14.7 (5.8 to 18.3)	0.8 (0.0 to 2.2)	0.017
**Duodenum**	D0.5cc	32.2 (5.4 to 33.5)	30.0 (4.1 to 32.7)	0.7 (0.1 to 1.2)	<0.001*
**(*n*** **=** **41)**	D5cc	25.7 (10.2 to 31.5)	23.2 (9.2 to 29.7)	0.6 (0.1 to 1.7)	<0.001*
	D10cc	20.3 (7.5 to 30.8)	19.4 (6.7 to 28.9)	0.6 (0.0 to 1.5)	<0.001*
**Stomach (n** **=** **41)**	D0.5cc	15.2 (5.2 to 27.7)	14.9 (5.3 to 28.7)	0.0 (−0.8 to 1.3)	0.462
	D5cc	13.2 (6.4 to 20.6)	13.3 (6.1 to 22.8)	0.3 (−2.3 to 1.7)	0.746
	D10cc	10.3 (4.3 to 17.6)	11.1 (5.4 to 17.7)	0.6 (−1.4 to 1.5)	0.886
	D50cc	2.0 (1.2 to 9.0)	2.6 (1.6 to 8.9)	0.0 (−0.6 to 0.2)	0.431
**R kidney (*n*** **=** **37)**	Dmean	4.2 (1.6 to 6.9)	4.0 (1.9 to 6.4)	0.0 (−0.2 to 0.2)	0.783
**L kidney (*n*** **=** **39)**	Dmean	4.4 (1.1 to 5.5)	4.3 (1.3 to 5.5)	0.0 (−0.1 to 0.3)	0.525
**Heart (*n*** **=** **11)**	D0.5cc	7.9 (5.4 to 15.7)	7.1 (5.2 to 15.1)	0.2 (0.0 to 1.0)	0.035
**Chest wall (n** **=** **11)**	D0.5cc	30.9 (18.8 to 36.5)	27.0 (17.6 to 35.5)	1.2 (0.9 to 1.6)	>0.999
**Oesophagus (n** **=** **11)**	D0.5cc	12.2 (7.9 to 22.1)	11.7 (7.2 to 21.8)	0.6 (−0.1 to 0.7)	0.269

### 3.3 Symptoms

Among the 40 patients with available baseline data, 40 symptoms were reported, with some patients reporting multiple, most commonly those with LAPC. The most common baseline symptoms reported were Grade (G) 1 fatigue (*n* = 14, 35.0%) and G1 diarrhoea (*n* = 7, 17.5%). Four G2 symptoms (fatigue and anorexia) and one G3 diarrhoea were also reported.

Post-treatment symptom data were unavailable for six patients: one patient withdrew from symptom collection, three had died (all LAPC), and two were too unwell for assessment due to advanced disease. Among the 35 evaluable patients at three months post-SABR, the most common symptoms were G1 fatigue (*n* = 16, 45.7%) and G1 abdominal pain (*n* = 14, 40.0%). Twelve G2 symptoms were reported in nine patients: fatigue (*n* = 7, 20.0%), diarrhoea (*n* = 3, 8.6%), anorexia (*n* = 1, 2.9%), and abdominal pain (*n* = 1, 2.9%). No ≥G3 acute symptoms were reported.

At 6 months, 30 subacute symptoms were recorded for 30 patients, most commonly G1 fatigue (*n* = 11, 36.7%), abdominal pain (*n* = 8, 26.7%), diarrhoea (*n* = 4, 13.3%), anorexia (*n* = 3, 10%), and bloating and nausea in one patient each (*n* = 3.3% respectively). Five G2 symptoms were reported: fatigue (*n* = 2, 6.7%), abdominal pain (*n* = 2, 6.7%), and diarrhoea (*n* = 1, 3.3%). No ≥G3 subacute symptoms were reported.

### 3.4 Survival and subsequent treatment following SABR

**Fig. 3:**
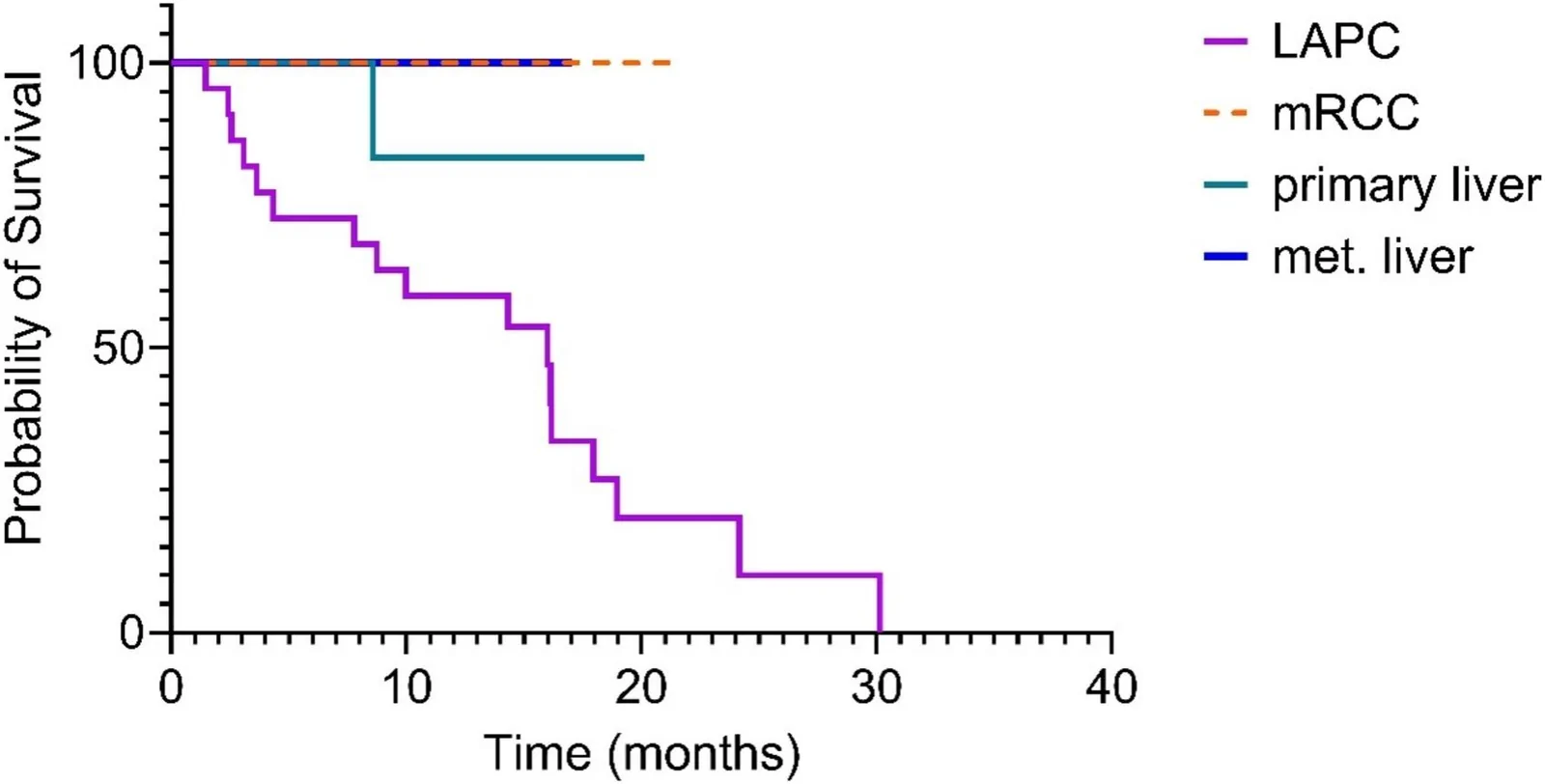
Kaplan–Meier survival curve for 38 patients included in the analysis: 22 patients with locally advanced pancreatic cancer (LAPC), five with metastatic renal cell carcinoma (mRCC), eight with primary liver tumours, and 3 with liver metastases. Three patients were lost to follow-up and therefore excluded from this analysis.

Three patients with LAPC (7.3%) with available follow-up records had died due to progressive disease within 3 months of SABR completion. At 6 months post-SABR, 21 of patients with LAPC (87.5%), all five with mRCC (100%), and all 11 patients who received liver SABR (100%) were alive. Kaplan-Meier analysis found that the median OS for LAPC was 16.0 months and was not reached for any other sub-groups (mRCC, primary/metastatic liver, Fig. 3). Subsequent systemic treatment within 6 months was recorded in four patients (Table 1).

### 3.5 Dose-side effect relationship

Thirty-five patients had data available for correlation testing between ΔCTCAE and AD to OARs at 3 months post-SABR. Weak positive, statistically significant, relationships were found between all three small bowel metrics (D0.5cc/D5cc/D10cc) and diarrhoea (r_s_ 0.4, *p* < 0.05, Table 3), indicating worsening of symptoms with increasing dose. A moderate positive relationship was also found between anorexia and stomach D50cc (r_s_ 0.4, 95% CI [0.0 to 0.6], *p* < 0.05). Weak negative associations between large bowel/duodenum AD and fatigue were found (Supplementary Table 2), suggesting no consistent dose-side effect relationship.

**Table 3:** Summary of univariable correlation analysis results with Spearman's rho (r_s_) and statistical *p*-value (*p*) for acute (3 month) and subacute (6 months) symptoms and accumulated dose to organs at risk (OARs) following SABR. Dose metrics include mean dose (Dmean), dose to 0.5 cc (D0.5cc), 10 cc (D10cc, and 50 cc (D50cc). Statistically significant results (*p* < 0.05) are indicated with an asterisk (*). Note: ΔCTCAE was calculated as follow-up minus baseline, therefore positive r_s_ values indicate worsening symptoms with increasing dose; negative r_s_ values indicate symptom improvement or less worsening with increasing dose. Full results are available in Supplementary Table 2.

Symptom	OAR (metric)	3 months					6 months				
		r_s_	95% CI			*p*	r_s_	95% CI			*p*
**Abdominal pain**	Liver (Dmean)	−0.2	−0.5	to	0.2	0.320	0.2	−0.2	to	0.5	0.232
	Large bowel (D0.5cc)	−0.4	−0.6	to	0.0	0.045*	−0.5	−0.7	to	−0.1	0.009*
	Large bowel (D5cc)	−0.3	−0.6	to	0.0	0.057	−0.4	−0.7	to	0.0	0.028*
	Large bowel (D10cc)	−0.2	−0.6	to	0.1	0.191	−0.4	−0.7	to	0.0	0.036*
	Duodenum (D0.5cc)	0.1	−0.3	to	0.4	0.621	0.3	−0.1	to	0.6	0.103
	Duodenum (D5cc)	0.1	−0.3	to	0.4	0.632	0.3	−0.1	to	0.6	0.174
	Duodenum (D10cc)	0.1	−0.3	to	0.4	0.621	0.2	−0.2	to	0.6	0.229
	Stomach (D0.5cc)	0.0	−0.4	to	0.3	0.988	0.2	−0.1	to	0.6	0.198
	Stomach (D5cc)	0.0	−0.4	to	0.3	0.842	0.2	−0.2	to	0.5	0.314
	Stomach (D10cc)	−0.1	−0.4	to	0.3	0.707	0.2	−0.2	to	0.5	0.261
	Stomach (D50cc)	0.0	−0.3	to	0.4	0.840	−0.1	−0.5	to	0.3	0.512
**Diarrhoea**	Small bowel (D0.5cc)	0.4	0.0	to	0.6	0.040*	0.4	0.1	to	0.7	0.019*
	Small bowel (D5cc)	0.4	0.0	to	0.6	0.029*	0.5	0.1	to	0.7	0.014*
	Small bowel (D10cc)	0.4	0.0	to	0.6	0.029*	0.5	0.1	to	0.7	0.014*
	Duodenum (D0.5cc)	0.2	−0.2	to	0.5	0.272	0.3	−0.1	to	0.6	0.170
	Duodenum (D5cc)	0.2	−0.1	to	0.5	0.222	0.2	−0.2	to	0.5	0.300
	Duodenum (D10cc)	0.2	−0.1	to	0.5	0.165	0.2	−0.1	to	0.6	0.191
**Nausea**	Small bowel (D0.5cc)	0.0	−0.4	to	0.3	0.305	−0.3	−0.6	to	0.1	0.081
	Small bowel (D5cc)	−0.1	−0.4	to	0.3	0.268	−0.3	−0.6	to	0.0	0.067
	Small bowel (D10cc)	−0.1	−0.4	to	0.3	0.256	−0.4	−0.6	to	0.0	0.054
	Duodenum (D0.5cc)	0.0	−0.4	to	0.3	0.298	−0.3	−0.6	to	0.1	0.176
	Duodenum (D5cc)	−0.1	−0.4	to	0.3	0.281	−0.3	−0.6	to	0.1	0.092
	Duodenum (D10cc)	−0.1	−0.4	to	0.3	0.270	−0.3	−0.6	to	0.1	0.092
	Stomach (D0.5cc)	−0.2	−0.5	to	0.2	0.174	−0.4	−0.6	to	0.0	0.052
	Stomach (D5cc)	−0.2	−0.5	to	0.1	0.136	−0.4	−0.7	to	0.0	0.042*
	Stomach (D10cc)	−0.2	−0.5	to	0.1	0.103	−0.4	−0.7	to	0.0	0.034*
	Stomach (D50cc)	−0.2	−0.5	to	0.2	0.162	−0.1	−0.5	to	0.3	0.627

Thirty patients were included in correlation testing between ΔCTCAE and OARs AD at 6-months post-SABR. Moderate, statistically significant, negative relationships were found between all large bowel dose metrics and abdominal pain at 6 months (r_s_ range − 0.4 to −0.5, 95% CI range [−0.1 to −0.7], *p* < 0.05). Moderate, statistically significant, relationships between large bowel D0.5cc/D5cc and fatigue were also found (r_s_ − 0.4 and − 0.5, respectively, *p* < 0.05, Supplementary Table 2). Weak, non-significant positive relationships were found between duodenum/stomach dose and both diarrhoea and abdominal pain at 6 months, further details in Table 3.

## 4 Discussion

This work introduces and applies a novel DIR-based dose accumulation pipeline for MR-guided SABR for liver and pancreatic tumours, designed to enable correlation of AD with clinical outcomes. Forty-one patients treated on an MR-Linac were included. Treatment was well tolerated, with no side effects exceeding G2. The most common acute side effects were G1 fatigue and abdominal pain, while G1 fatigue was the most common subacute effect. Overall, OAR AD was typically lower than planned. This pipeline demonstrated the feasibility of relating AD to changes in symptom burden from baseline, establishing a foundation for future hypothesis-driven analyses of dose-symptom relationships.

A key strength of this single-institution study is the development and prospective application of a reproducible DIR-based dose accumulation workflow in a consecutive, ‘real-world’ cohort, rather than a highly selected trial population. This pragmatic design supports relevance to routine clinical practice, although loss to follow-up and incomplete data may influence interpretation of downstream outcome signals. Importantly, the methodological steps described here can be implemented at other centres, supporting external adoption and validation of the workflow. As this is a contour-driven DIR approach, performance inherently depends on delineation accuracy, with small contour discrepancies potentially propagating into local dose uncertainties. Prior work from our institution (unpublished) indicates low intra-observer variation, with median mDTA of 0.9 mm (IQR 0.7–1.1 mm). Inter-observer variation was not quantified in this work. It should also be noted that subsequent systemic therapy may have influenced symptom reporting. Therefore, associations between AD and outcomes should be interpreted as exploratory, particularly given the limited cohort size and number of symptom events precluding meaningful site-specific analyses. While the present dataset was insufficient to robustly evaluate subgroup cohorts separately, the workflow provides a framework for future studies incorporating larger cohorts and more detailed clinical outcome data.

DIR performance metrics from this study compare favourably with previous liver SABR reports for several OARs, including the stomach (DSC 0.94 ± 0.15 *versus* 0.88 ± 0.12) [19]. Larger observed HD values likely reflect genuine inter-fraction anatomical variation, such as fasting-related changes in stomach size and variation in craniocaudal imaging FOV, which may have limited impact on volume overlap but can generate substantial point-wise deviations. These findings highlight known challenges of DIR in highly deformable GI OARs [19], [20], and the need for cautious interpretation of AD estimates for these structures. Although these metrics assess contour agreement, they do not directly validate voxel-level deformation accuracy and are not entirely independent because the structures were used to constrain the DVFs. TRE could not be assessed in this study, representing an additional limitation when interpreting AD estimates in high-dose gradient regions. From a practical standpoint, a summed DVH approach may remain appropriate and intentionally conservative when OAR doses are close to tolerance, accepting potential overestimation of dose differences [19]. Systematic characterisation of these uncertainties is an important next step for refining this pipeline and supporting its use in scenarios such as re-irradiation or adaptive strategies guided by cumulative dose.

In this cohort, accumulated OAR doses remained within clinical constraints. Although several statistically significant differences between planned and AD were detected, their absolute magnitudes were small and unlikely to be clinically meaningful. They may partly reflect DIR-related smoothing of high-dose regions not captured by contour-based QA [21]. Lower AD may also reflect daily ATS plans themselves, including conservative online re-optimisation and prioritisation of OAR constraints, rather than DIR-based dose accumulation alone. The value of this analysis therefore is to demonstrate the behaviour of the accumulation pipeline rather than defining new tolerance thresholds. Unlike Regnery et al. [19], who reported stomach and intestinal D0.5cc exceeding reference plan doses by up to 18.1 Gy and 19.7 Gy, respectively, our study estimated lower-than-planned AD for these OARs. This suggests that local implementation choices and patient preparation can meaningfully influence AD patterns and should be captured in future multi-centre work.

Prior series of MR-guided adaptive pancreatic and liver SABR have reported predominantly ≤ G2 side effects, with occasional ≥ G3 events at higher doses [22], [23]. Consistent with these reports, our cohort, which included patients with pre-existing symptom burden from prior systemic/local therapies, experienced no acute or subacute side effects > G2, supporting acceptable tolerability in routine practice. As follow-up was limited to 6 months, capturing acute/subacute symptoms only, and subsequent systemic therapy may also have influenced reported symptoms, no conclusions regarding late toxicity can be drawn. The moderate positive correlation between small bowel AD and acute diarrhoea is consistent with previously reported dose–symptom relationships and illustrates how this pipeline can generate hypothesis-generating signals for dedicated outcome studies [24], [25]. However, because the primary objective of this work was establishing feasibility, these findings should be interpreted as exploratory rather than definitive.

Because most existing normal-tissue complication probability and dose–response models are derived from reference plan dosimetry, DIR-based dose accumulation could refine these models using AD [10]. The workflow presented here is intended as such a platform: our centre is extending this methodology across multiple anatomical sites to explore associations between AD, clinical outcomes, treatment-related symptoms, and laboratory and imaging biomarkers. Prospective evaluation in larger, independent cohorts will be required to validate the robustness and generalisability of the pipeline, but this study establishes the necessary infrastructure and demonstrates feasibility in a real-world MR-guided SABR programme.

In conclusion, evaluating relationships between DIR-based AD and side effects was feasible in this MR-guided SABR cohort. Exploratory relationships between OAR AD and side effects were observed, but larger studies are needed before these findings can support reliable treatment personalisation.

## CRediT authorship contribution statement

**Rekaya Shabbir:** Writing – original draft, Project administration, Investigation, Formal analysis. **Agnieszka Zakacz:** Writing – original draft, Investigation, Formal analysis. **Ruksana Sivakaran:** Writing – review & editing, Investigation. **Eliana Vasquez Osorio:** Writing – review & editing, Methodology, Conceptualization. **Marianna Theodoulou:** Writing – review & editing, Investigation. **Zainul Kapacee:** Writing – review & editing, Investigation. **Ananya Choudhury:** Writing – review & editing, Funding acquisition. **Laura J. Forker:** Writing – review & editing, Conceptualization. **Ganesh Radhakrishna:** Writing – review & editing, Conceptualization. **Cynthia L. Eccles:** Writing – review & editing, Methodology, Funding acquisition, Conceptualization. **Mairead Daly:** Writing – original draft, Visualization, Supervision, Project administration, Methodology, Investigation, Formal analysis, Data curation, Conceptualization.

## Funding

Rekaya Shabbir and Mairead Daly are supported by Cancer Research UK RadNet Manchester [C1994/A28701]. Eliana Vasquez Osorio is supported by 10.13039/100014013UK Research and Innovation [UKRI2334]. Ananya Choudhury and Cynthia Eccles are supported by 10.13039/100014653NIHR Manchester Biomedical Research Centre (NIHR203308). This work is also supported by Cancer Research UK Manchester Centre (CTRQQR-2021\100010).

## Declaration of competing interest

The authors declare the following financial interests/personal relationships which may be considered as potential competing interests:(Mairead Daly reports financial support was provided by Cancer Research UK. Rekaya Shabbir reports financial support was provided by Cancer Research UK. Eliana Vasquez Osorio reports financial support was provided by UK Research and Innovation. Ananya Choudhury reports was provided by NIHR Manchester Biomedical Research Centre. Cynthia Eccles reports was provided by NIHR Manchester Biomedical Research Centre. This work is also supported by reports financial support was provided by Cancer Research UK. If there are other authors, they declare that they have no known competing financial interests or personal relationships that could have appeared to influence the work reported in this paper.)
