# Supplementary material for: Feasibility of dose accumulation for assessing treatment-related side effects in MR-guided abdominal SABR: A single-institution study
Source: Clin Transl Radiat Oncol. 2026 Sep 2;61:101277. doi: 10.1016/j.ctro.2026.101277 (PMC13571963; doi:10.1016/j.ctro.2026.101277)
Supplement: Supplementary file 1 [file mmc1.docx]

**Supplementary Figure 1.** Schema of planning computed tomography (CT) and magnetic resonance imaging (MRI), as well as delineation details, used for reference plan generation. EBH = exhale breath hold, bFFE = balanced steady-state free precession gradient echo, SPAIR = spectral attenuated inversion recovery, TE = echo time, TR = relaxation time, ms = milliseconds, FOV = field of view, GTV = gross tumour volume, ITV = internal target volume, PTV = planning target volume, PRV = planning organ at risk volume, MDT = multidisciplinary team.

**Supplementary Table 1.** Population median (interquartile range − IQR) in millimetres (mm) of similarity metrics between contoured and deformably mapped structures across all patients and fractions. mDTA = mean distance to agreement, number of fractions (#) where mDTA < 2mm, HD = maximum Hausdorff distance, DSC = Dice similarity coefficient. Note: DSC is presented here to two decimal places for granularity.

|  |  | | **mDTA** | | | | | | | | | **HD** | | | | | | | | | | | | **DSC** | | | | | | | | | | |
| --- | --- | --- | --- | --- | --- | --- | --- | --- | --- | --- | --- | --- | --- | --- | --- | --- | --- | --- | --- | --- | --- | --- | --- | --- | --- | --- | --- | --- | --- | --- | --- | --- | --- | --- |
|  |  | | **Median** | | | **IQR** | | | | | | **Median** | | **IQR** | | | | | | | | | | **Median** | | | **IQR** | | | | | | | |
|  | GTV | | 0.1 | | | 0.1 | | to | | 0.2 | | 2.1 | | 1.5 | | | to | | 2.8 | | | | 0.98 | | | 0.97 | | | | to | | 0.98 | |  |
|  | Liver | | 0.1 | | | 0.1 | | to | | 0.2 | | 4.6 | | 3.3 | | | to | | 7.3 | | | | 0.99 | | | 0.99 | | | | to | | 1.00 | |  |
|  | Large Bowel | | 1.2 | | | 0.4 | | to | | 3.1 | | 28.2 | | 13.2 | | | to | | 40.6 | | | | 0.91 | | | 0.82 | | | | to | | 0.97 | |  |
|  | Small bowel | | 2.3 | | | 0.8 | | to | | 4.2 | | 26.8 | | 15.6 | | | to | | 36.5 | | | | 0.84 | | | 0.66 | | | | to | | 0.94 | |  |
|  | Duodenum | | 0.1 | | | 0.1 | | to | | 0.4 | | 4.2 | | 2.4 | | | to | | 12.7 | | | | 0.98 | | | 0.96 | | | | to | | 0.98 | |  |
|  | Stomach | | 0.1 | | | 0.1 | | to | | 0.3 | | 3.3 | | 2.2 | | | to | | 7.3 | | | | 0.99 | | | 0.98 | | | | to | | 0.99 | |  |
|  | R kidney | | 0.1 | | | 0.1 | | to | | 0.1 | | 2.0 | | 1.5 | | | to | | 2.6 | | | | 0.99 | | | 0.99 | | | | to | | 1.00 | |  |
|  | L kidney | | 0.1 | | | 0.1 | | to | | 0.1 | | 2.2 | | 1.5 | | | to | | 3.2 | | | | 0.99 | | | 0.99 | | | | to | | 1.00 | |  |
|  | Heart | | 0.1 | | | 0.1 | | to | | 0.1 | | 2.0 | | 1.5 | | | to | | 2.2 | | | | 1.00 | | | 1.00 | | | | to | | 1.00 | |  |
|  | Chestwall | | 0.1 | | | 0.0 | | to | | 0.1 | | 2.5 | | 2.0 | | | to | | 3.2 | | | | 1.00 | | | 0.99 | | | | to | | 1.00 | |  |
|  | Oesophagus | | 0.1 | | | 0.1 | | to | | 0.1 | | 1.5 | | 1.0 | | | to | | 2.0 | | | | 0.98 | | | 0.98 | | | | to | | 0.99 | |  |
|  |  | |  | | |  | |  | | |  |  | | |  | | | | |  | |  | |  | | | |  | | | | |  |  |
|  | |  | |  |  | |  | |  | | | |  | | |  | |  | | |  | | | |  | | | |  | |  |  |  |  |

**Supplementary Table 2:** Full univariable correlation analysis results with Spearman’s rho (r_s_) and statistical p−value (*p*) for acute (3 month) and subacute (6 months) symptoms and accumulated dose to organs at risk (OARs) following SABR. Dose metrics include mean dose (Dmean), dose to 0.5cc (D0.5cc), 10cc (D10cc, and 50cc (D50cc). Statistically significant results (*p* < 0.05) are indicated with an asterisk (*).

| **Symptom** | **OAR (metric)** | **3 months** | | | | | **6 months** | | | | |
| --- | --- | --- | --- | --- | --- | --- | --- | --- | --- | --- | --- |
|  |  | **r** | **95% CI** | | | ***p*** | **r** | **95% CI** | | | ***p*** |
| **Abdominal pain** | Liver (Dmean) | −0.2 | −0.5 | to | 0.2 | 0.320 | 0.2 | −0.2 | to | 0.5 | 0.232 |
|  | Large bowel (D0.5cc) | −0.4 | −0.6 | to | 0.0 | 0.045* | −0.5 | −0.7 | to | −0.1 | 0.009* |
|  | Large bowel (D5cc) | −0.3 | −0.6 | to | 0.0 | 0.057 | −0.4 | −0.7 | to | 0.0 | 0.028* |
|  | Large bowel (D10cc) | −0.2 | −0.6 | to | 0.1 | 0.191 | −0.4 | −0.7 | to | 0.0 | 0.036* |
|  | Small bowel (D0.5cc) | −0.1 | −0.4 | to | 0.3 | 0.725 | 0.2 | −0.2 | to | 0.5 | 0.422 |
|  | Small bowel (D5cc) | −0.1 | −0.4 | to | 0.3 | 0.661 | 0.2 | −0.2 | to | 0.5 | 0.429 |
|  | Small bowel (D10cc) | −0.1 | −0.4 | to | 0.3 | 0.621 | 0.2 | −0.2 | to | 0.5 | 0.393 |
|  | Duodenum (D0.5cc) | 0.1 | −0.3 | to | 0.4 | 0.621 | 0.3 | −0.1 | to | 0.6 | 0.103 |
|  | Duodenum (D5cc) | 0.1 | −0.3 | to | 0.4 | 0.632 | 0.3 | −0.1 | to | 0.6 | 0.174 |
|  | Duodenum (D10cc) | 0.1 | −0.3 | to | 0.4 | 0.621 | 0.2 | −0.2 | to | 0.6 | 0.229 |
|  | Stomach (D0.5cc) | 0.0 | −0.3 | to | 0.3 | 0.988 | 0.2 | −0.1 | to | 0.6 | 0.198 |
|  | Stomach (D5cc) | 0.0 | −0.4 | to | 0.3 | 0.842 | 0.2 | −0.2 | to | 0.5 | 0.314 |
|  | Stomach (D10cc) | −0.1 | −0.4 | to | 0.3 | 0.707 | 0.2 | −0.2 | to | 0.5 | 0.261 |
|  | Stomach (D50cc) | 0.0 | −0.3 | to | 0.4 | 0.840 | −0.1 | −0.5 | to | 0.3 | 0.512 |
| **Diarrhoea** | Liver (Dmean) | −0.2 | −0.5 | to | 0.1 | 0.236 | 0.1 | −0.3 | to | 0.4 | 0.634 |
|  | Large bowel (D0.5cc) | 0.0 | −0.3 | to | 0.4 | 0.801 | 0.0 | −0.4 | to | 0.4 | 0.929 |
|  | Large bowel (D5cc) | 0.0 | −0.4 | to | 0.3 | 0.976 | 0.1 | −0.3 | to | 0.4 | 0.722 |
|  | Large bowel (D10cc) | 0.0 | −0.4 | to | 0.4 | 0.974 | 0.1 | −0.3 | to | 0.5 | 0.685 |
|  | Small bowel (D0.5cc) | 0.4 | 0.0 | to | 0.6 | 0.040* | 0.4 | 0.1 | to | 0.7 | 0.019* |
|  | Small bowel (D5cc) | 0.4 | 0.0 | to | 0.6 | 0.029* | 0.5 | 0.1 | to | 0.7 | 0.014* |
|  | Small bowel (D10cc) | 0.4 | 0.0 | to | 0.6 | 0.029* | 0.5 | 0.1 | to | 0.7 | 0.014* |
|  | Duodenum (D0.5cc) | 0.2 | −0.2 | to | 0.5 | 0.272 | 0.3 | −0.1 | to | 0.6 | 0.170 |
|  | Duodenum (D5cc) | 0.2 | −0.1 | to | 0.5 | 0.222 | 0.2 | −0.2 | to | 0.5 | 0.300 |
|  | Duodenum (D10cc) | 0.2 | −0.1 | to | 0.5 | 0.165 | 0.2 | −0.1 | to | 0.6 | 0.191 |
|  | Stomach (D0.5cc) | 0.1 | −0.3 | to | 0.4 | 0.569 | 0.3 | −0.1 | to | 0.6 | 0.086 |
|  | Stomach (D5cc) | 0.1 | −0.3 | to | 0.4 | 0.742 | 0.2 | −0.1 | to | 0.6 | 0.204 |
|  | Stomach (D10cc) | 0.0 | −0.3 | to | 0.3 | 0.988 | 0.3 | −0.1 | to | 0.6 | 0.165 |
|  | Stomach (D50cc) | 0.0 | −0.4 | to | 0.3 | 0.973 | 0.2 | −0.2 | to | 0.5 | 0.359 |
| **Nausea** | Liver (Dmean) | −0.1 | −0.5 | to | 0.2 | 0.222 | 0.1 | −0.3 | to | 0.4 | 0.695 |
|  | Large bowel (D0.5cc) | −0.2 | −0.5 | to | 0.2 | 0.166 | −0.1 | −0.5 | to | 0.3 | 0.557 |
|  | Large bowel (D5cc) | −0.2 | −0.5 | to | 0.2 | 0.159 | −0.1 | −0.5 | to | 0.3 | 0.557 |
|  | Large bowel (D10cc) | −0.1 | −0.4 | to | 0.3 | 0.266 | 0.1 | −0.3 | to | 0.5 | 0.617 |
|  | Small bowel (D0.5cc) | 0.0 | −0.4 | to | 0.3 | 0.305 | −0.3 | −0.6 | to | 0.1 | 0.081 |
|  | Small bowel (D5cc) | −0.1 | −0.4 | to | 0.3 | 0.268 | −0.3 | −0.6 | to | 0.0 | 0.067 |
|  | Small bowel (D10cc) | −0.1 | −0.4 | to | 0.3 | 0.256 | −0.4 | −0.6 | to | 0.0 | 0.054 |
|  | Duodenum (D0.5cc) | 0.0 | −0.4 | to | 0.3 | 0.298 | −0.3 | −0.6 | to | 0.1 | 0.176 |
|  | Duodenum (D5cc) | −0.1 | −0.4 | to | 0.3 | 0.281 | −0.3 | −0.6 | to | 0.1 | 0.092 |
|  | Duodenum (D10cc) | −0.1 | −0.4 | to | 0.3 | 0.270 | −0.3 | −0.6 | to | 0.1 | 0.092 |
|  | Stomach (D0.5cc) | −0.2 | −0.5 | to | 0.2 | 0.174 | −0.4 | −0.6 | to | 0.0 | 0.052 |
|  | Stomach (D5cc) | −0.2 | −0.5 | to | 0.1 | 0.136 | −0.4 | −0.7 | to | 0.0 | 0.042* |
|  | Stomach (D10cc) | −0.2 | −0.5 | to | 0.1 | 0.103 | −0.4 | −0.7 | to | 0.0 | 0.034* |
|  | Stomach (D50cc) | −0.2 | −0.5 | to | 0.2 | 0.162 | −0.1 | −0.5 | to | 0.3 | 0.627 |
| **Anorexia** | Liver (Dmean) | 0.2 | −0.1 | to | 0.5 | 0.242 | 0.3 | −0.1 | to | 0.6 | 0.097 |
|  | Large bowel (D0.5cc) | 0.0 | −0.4 | to | 0.3 | 0.985 | 0.1 | −0.3 | to | 0.5 | 0.664 |
|  | Large bowel (D5cc) | 0.0 | −0.3 | to | 0.4 | 0.885 | 0.1 | −0.3 | to | 0.5 | 0.486 |
|  | Large bowel (D10cc) | −0.1 | −0.5 | to | 0.2 | 0.470 | 0.1 | −0.3 | to | 0.5 | 0.470 |
|  | Small bowel (D0.5cc) | −0.1 | −0.4 | to | 0.3 | 0.742 | −0.1 | −0.4 | to | 0.3 | 0.675 |
|  | Small bowel (D5cc) | −0.1 | −0.4 | to | 0.3 | 0.747 | 0.0 | −0.4 | to | 0.3 | 0.867 |
|  | Small bowel (D10cc) | 0.0 | −0.4 | to | 0.3 | 0.807 | 0.0 | −0.4 | to | 0.3 | 0.802 |
|  | Duodenum (D0.5cc) | 0.1 | −0.3 | to | 0.4 | 0.745 | 0.0 | −0.4 | to | 0.4 | >0.9999 |
|  | Duodenum (D5cc) | 0.0 | −0.3 | to | 0.4 | 0.898 | −0.1 | −0.4 | to | 0.3 | 0.626 |
|  | Duodenum (D10cc) | 0.0 | −0.3 | to | 0.4 | 0.868 | −0.1 | −0.4 | to | 0.3 | 0.626 |
|  | Stomach (D0.5cc) | 0.1 | −0.2 | to | 0.4 | 0.500 | 0.1 | −0.3 | to | 0.4 | 0.626 |
|  | Stomach (D5cc) | 0.2 | −0.2 | to | 0.5 | 0.298 | 0.0 | −0.3 | to | 0.4 | 0.871 |
|  | Stomach (D10cc) | 0.3 | −0.1 | to | 0.6 | 0.123 | 0.0 | −0.3 | to | 0.4 | 0.808 |
|  | Stomach (D50cc) | 0.4 | 0.0 | to | 0.6 | 0.036* | 0.1 | −0.3 | to | 0.5 | 0.557 |
| **Fatigue** | Liver (Dmean) | 0.1 | −0.2 | to | 0.4 | 0.492 | 0.3 | −0.1 | to | 0.6 | 0.163 |
|  | Large bowel (D0.5cc) | −0.3 | −0.6 | to | 0.0 | 0.057 | −0.5 | −0.7 | to | −0.1 | 0.010* |
|  | Large bowel (D5cc) | −0.4 | −0.6 | to | 0.0 | 0.034* | −0.4 | −0.7 | to | 0.0 | 0.033* |
|  | Large bowel (D10cc) | −0.4 | −0.7 | to | 0.0 | 0.031* | −0.3 | −0.6 | to | 0.1 | 0.136 |
|  | Small bowel (D0.5cc) | −0.1 | −0.4 | to | 0.3 | 0.573 | −0.1 | −0.5 | to | 0.3 | 0.471 |
|  | Small bowel (D5cc) | −0.1 | −0.5 | to | 0.2 | 0.487 | −0.2 | −0.5 | to | 0.2 | 0.366 |
|  | Small bowel (D10cc) | −0.1 | −0.5 | to | 0.2 | 0.414 | −0.2 | −0.5 | to | 0.2 | 0.300 |
|  | Duodenum (D0.5cc) | −0.2 | −0.5 | to | 0.2 | 0.310 | 0.0 | −0.4 | to | 0.4 | 0.995 |
|  | Duodenum (D5cc) | −0.3 | −0.6 | to | 0.1 | 0.133 | −0.1 | −0.4 | to | 0.3 | 0.628 |
|  | Duodenum (D10cc) | −0.3 | −0.6 | to | 0.1 | 0.137 | −0.1 | −0.5 | to | 0.3 | 0.610 |
|  | Stomach (D0.5cc) | 0.0 | −0.4 | to | 0.3 | 0.808 | 0.0 | −0.3 | to | 0.4 | 0.886 |
|  | Stomach (D5cc) | 0.0 | −0.4 | to | 0.3 | 0.840 | 0.0 | −0.4 | to | 0.4 | 0.979 |
|  | Stomach (D10cc) | 0.0 | −0.3 | to | 0.3 | 0.992 | 0.0 | −0.4 | to | 0.4 | 0.912 |
|  | Stomach (D50cc) | 0.1 | −0.3 | to | 0.4 | 0.684 | 0.1 | −0.3 | to | 0.4 | 0.745 |
|  | | | | | | | | | | | |
|  |  |  |  |  |  |  |  |  |  |  |  |
